# The relationship between depression and academic burnout among undergraduate students majoring in eldercare services: a moderated mediation model

**DOI:** 10.3389/fpsyg.2025.1632556

**Published:** 2025-08-18

**Authors:** Yingjie Lin, Siyu Pan, Jianyao He, Qi Cheng, Hongmin Wei, Li Zhao

**Affiliations:** 1 School of Geriatrics and Elderly Care Industry, Shenyang Medical College, Shenyang, Liaoning, China; 2 Division of Academic Affairs, Kunming Medical University, Kunming, Yunnan, China; 3 School of Health Industry, Anshan Normal University, Anshan, Liaoning, China; 4 Department of Nursing, Beijing Health Vocational College, Beijing, China

**Keywords:** depression, self-efficacy, academic burnout, social support, moderated mediation

## Abstract

**Objectives:**

To explore the relationship between depression, self-efficacy, academic burnout, and social support among undergraduate students majoring in eldercare services in China.

**Methods:**

A cross-sectional study was carried out among 957 students majoring in eldercare services across four provinces of China. The study employed questionnaires based on the Center for Epidemiological Studies Depression Scale, General Self-Efficacy Scale, Learning Burnout Scale, and Perceived Social Support Scale. Using SPSS 22.0 for descriptive analysis and Pearson correlation analysis, and constructed a moderated mediation model using PROCESS.

**Results:**

The findings reveal that 26.92% of students suffer from academic burnout. Depression is significantly negatively correlated with self-efficacy, positively correlated with academic burnout. Self-efficacy is negatively correlated with academic burnout. The results of the moderated mediation model showed that the mediating effect value of self-efficacy on depression and academic burnout was 0.139, accounting for 19.02% of the total effect. The interaction term between depression and social support has a negative effect on self-efficacy β = −0.203, and a positive effect on academic burnout *β* = 0.106.

**Conclusion:**

There is a positive correlation between depression and academic burnout among undergraduate students majoring in eldercare services. Self-efficacy partially mediates the relationship between depression and academic burnout, while social support plays a moderating role.

## 1 Introduction

With the acceleration of population aging and the deepening implementation of the aging strategy, the professional talent gap faced by the elderly care industry is becoming increasingly prominent. According to the practical needs of elderly health care, the discipline of elderly health management has rapidly developed with the support of Chinese policies. At present, Chinese elderly care related majors mainly include health services and management, elderly care services and management, labor and social security, laying a solid foundation for building a professional and standardized team of elderly care service talents.

The coexistence of academic burnout among college students has emerged as a public health challenge in the realm of global higher education. A meta-analysis of 42 studies involving 26,824 evaluations revealed an overall prevalence rate of 37.23% for job burnout among medical students (Almutairi et al., 2022). Compared with students in other specialties, those in the elderly care service profession are frequently exposed to the physiological decline, chronic diseases, disabilities, dementia, and terminal illnesses of the elderly population. Consequently, they are continually confronted with negative concepts such as the difficulty in curing diseases and the inevitability of death. This persistent exposure may lead to emotional exhaustion and a sense of helplessness, which can subsequently trigger depressive symptoms, reduce learning motivation, and contribute to academic burnout. Nevertheless, there has been scant research into the psychological wellbeing and academic burnout of undergraduates within this nascent discipline. The occurrence of academic burnout is often related to the interaction of multiple psychological and social factors. Therefore, this study conducts in-depth research on the relationship between depression, self-efficacy, social support, and academic burnout among undergraduate students majoring in eldercare services.

Depression is a negative psychological state characterized by sustained low mood, loss of interest, cognitive decline, and physical symptoms. Often accompanied by anxiety, helplessness, and behavioral withdrawal. College students, as the main population aged 18–24, have a higher risk of experiencing depression. According to relevant research, the detection rate of depression among Chinese college students is 31%, with a detection rate of 8% for severe depression (Zhang et al., 2023). Depressive emotions not only affect students’ physical and mental health, but also have negative effects on academic performance, learning efficiency, interpersonal communication, and social function, and even lead to suicide, bringing a heavy burden to families and society (Holm-Hadulla et al., 2023). Studies have shown that depression is significantly negatively correlated with self-efficacy and social support, and indirectly affects mental health through emotional instability and difficulty making choices (Marufi et al., 2024).

Academic burnout is a three-dimensional syndrome encompassing emotional exhaustion, academic alienation, and diminished sense of achievement, stemming from prolonged academic pressure. Its specific manifestations include physical and mental fatigue, resistance to learning, and self-denial of academic achievements (Bell and Kozlowski, 2002). Studies have revealed that the prevalence of severe burnout among Chinese adolescents stands at 23.7%, with a positive correlation observed between burnout and both anxiety and depression. Notably, academic burnout significantly impacts depression, exhibiting notable gender differences, where males exhibit significantly higher path coefficients than females (Sun et al., 2024). Furthermore, depressive symptoms and academic burnout tend to mutually reinforce each other over time, with this interaction being more pronounced among females (Zhang et al., 2024). Consequently, it can be deduced that depression also exerts a positive influence on academic burnout. Relevant research underscores that excessive academic burdens constitute a significant source of mental health issues among college students.

Self-efficacy refers to an individual’s level of confidence in their ability to successfully complete a specific task or behavior. It was proposed by psychologist Albert Bandura based on social cognitive theory and is one of the core concepts in social cognitive theory. It emphasizes how an individual’s belief in their own abilities affects their behavior, motivation, and emotions (Bandura, 1977). Studies indicate that individuals with high self-efficacy are inclined to confront stressors with a positive mindset and confidence, thereby decreasing levels of anxiety and depression (Zhang et al., 2025). Self-efficacy indirectly enhances mental health by modulating emotional responses and selecting positive coping strategies (Liu et al., 2024). Numerous studies have revealed a significant negative correlation between self-efficacy and academic burnout, primarily by reducing test anxiety and improving peer relationships to bolster psychological resilience, thus indirectly mitigating academic burnout (Zhu et al., 2023).

Social support refers to the extent to which an individual perceives the availability and willingness of assistance that can be obtained from others. On one hand, studies have demonstrated that social support exerts a regulatory effect by influencing health-promoting behaviors through self-efficacy, thereby mitigating depression (Park and Lee, 2023). When individuals experience depressive emotions, emotional care and practical assistance from family, school, or peers can alleviate feelings of loneliness and helplessness, reduce the negative impact of these emotions on self-perceived ability, and enable individuals to view their value and potential more objectively, thus maintaining or enhancing self-efficacy. Therefore, this study posits that an increase in students’ level of social support may buffer the negative impact of depression on self-efficacy. On the other hand, social support can effectively alleviate the adverse effects of external stressors, reduce negative emotions, ease psychological conflicts, and thereby contribute to the maintenance of mental and physical health. Relevant studies have shown that individuals with high levels of social support are better able to cope with stress, develop effective coping strategies, and consequently experience lower levels of psychological stress and a reduced likelihood of academic burnout (Ye et al., 2021; Yu et al., 2024; De Risio et al., 2024). Therefore, higher levels of social support may serve to moderate the relationship between depression and academic burnout among students.

**FIGURE 1:**
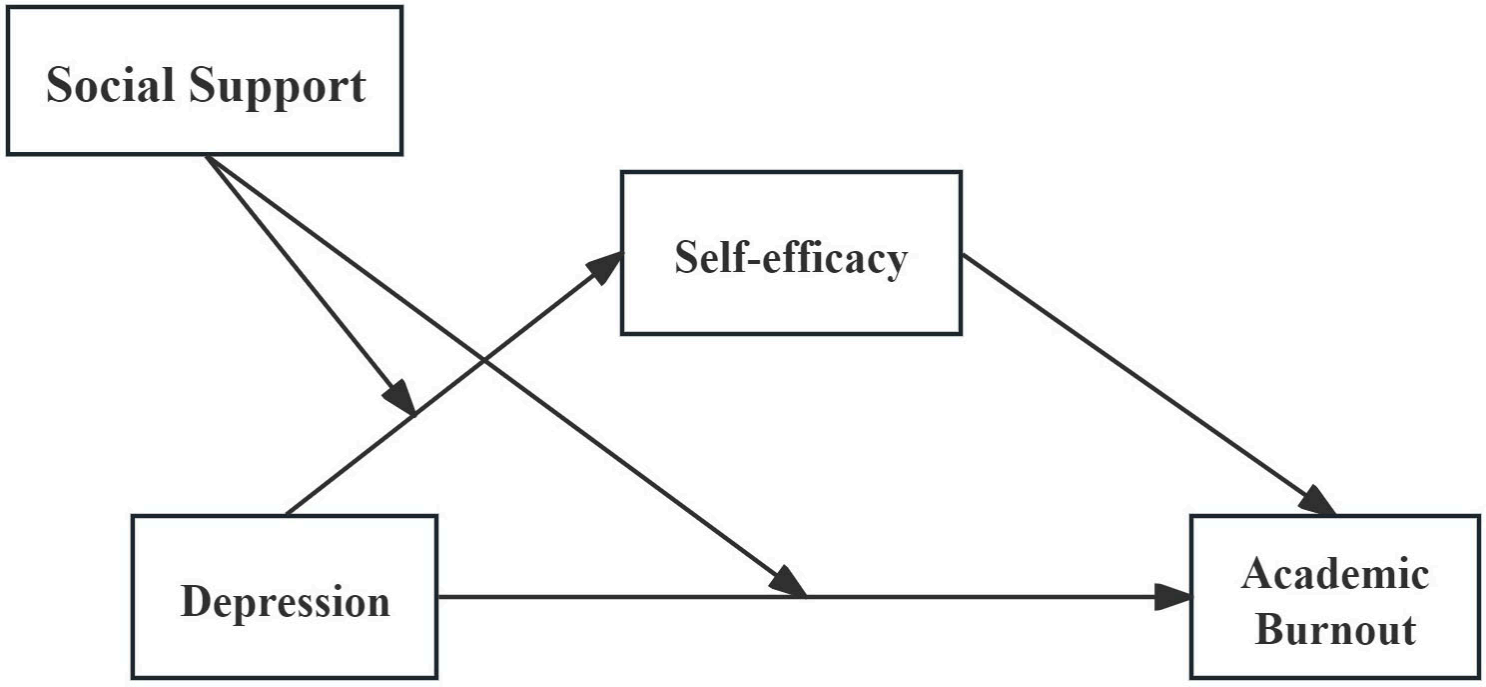
Research hypothesis model diagram.

Social cognitive theory holds that human behavior is the result of the interaction between individuals, society, and behavior. Human agency arises from the interaction between internal factors such as cognition, emotion, or biology, behavioral patterns, and environmental influences. Therefore, this study takes undergraduate students majoring in eldercare services as the research object, proposes the following four hypotheses, constructs a moderated mediation model between depression, self-efficacy, academic burnout, and social support, and explores the influencing factors of learning fatigue, which helps prevent and improve the problem of learning fatigue among undergraduate students majoring in eldercare services. It has important theoretical basis and practical value for promoting their professional knowledge learning, improving students’ mental health, providing high-quality talents for the elderly care service industry, and promoting the high-level development of the elderly care service industry (Figure 1).

Hypothesis 1: There exists a positive correlation between depression and academic burnout.
Hypothesis 2: Self-efficacy serves as a mediator in the relationship between academic burnout and depression.
Hypothesis 3: Social support moderates the relationship between depression and self-efficacy.
Hypothesis 4: Social support also moderates the relationship between depression and academic burnout.

## 2 Materials and methods

### 2.1 Study design and research subjects

This study is a cross-sectional study that used cluster sampling to select 957 undergraduate students majoring in eldercare services from four provinces in China, Liaoning, Shandong, Guangxi, and Yunnan, from September to November 2024. The samples for this study were selected from four geographical regions in China: Liaoning Province represents the Northeast region, Shandong Province represents East China, Guangxi Province represents South China, and Yunnan Province represents the Southwest region. These provinces encompass a broad spectrum of geographical, economic, and social diversity, ranging from coastal to inland areas and from developed to developing regions, thereby reflecting the key geographical, economic, and social characteristics of China. A questionnaire survey was conducted on their academic burnout, self-efficacy, depression, and social support. This research was conducted using the “Questionnaire Star” platform. This platform is a widely popular, highly functional and personalized questionnaire design system that can be used to publish instructions and questionnaires. The researchers clearly explained the purpose of data collection, anonymity and confidentiality measures on the questionnaire’s homepage to the participants, and obtained their informed consent. In this study, questionnaires with incomplete responses, identical answer patterns, or response times shorter than 150 s were excluded. A total of 780 valid questionnaires were collected, resulting in a response rate of 81.5%.

### 2.2 Research instruments

#### 2.2.1 Demographic characteristics of participants

This survey collects demographic information including gender, age, school, grade, whether they are the only child, parents’ educational level, scholarship status, student leadership position, daily exercise duration, daily sleep duration, smoking status, and parenting style.

#### 2.2.2 Center for Epidemiological Studies Depression Scale (CES-D)

The CES-D, developed by Radloff (1977), was employed in this study. This scale comprises 20 items and covers four dimensions: depressive affect, positive affect, somatic symptoms and activity retardation, and interpersonal difficulties. It uses a four-point Likert scale scoring method, ranging from “1” (not or rarely at all) to “4” (most or all of the time). The total score is the sum of all items, and ranges between 20 and 80. A total score < 15 indicates no depressive symptoms; scores between 16 and 19 suggest possible depressive symptoms; and scores > 20 indicate definite depressive symptoms. Compared with other scales used to measure depression, the CES-D is widely applicable in epidemiological surveys of the general population and can be self-administered by respondents, requiring only a short time to complete. In this study, the scale demonstrated excellent reliability and validity, with a Cronbach’s α coefficient of 0.939.

#### 2.2.3 General Self-Efficacy Scale (GSES)

The General Self-Efficacy Scale (GSES), developed by Schwarzer and Aristi (1997) was utilized in this study. This single-dimensional scale comprises 10 items and employs a four-point Likert scoring method, where scores range from “1” (completely correct) to “4” (completely incorrect). Each item is scored from 1 to 4, with higher scores indicating stronger self-efficacy. The total score ranges from 10 to 40, with higher scores reflecting greater self-efficacy (Schwarzer and Aristi, 1997). Compared with other scales, the GSES assesses an individual’s general belief in their overall ability rather than task-specific efficacy, and its 10-item structure imposes minimal burden on students during completion. In this study, the scale demonstrated excellent reliability and validity, with a Cronbach’s α coefficient of 0.958.

#### 2.2.4 Learning Burnout Scale (LBS)

The academic burnout scale employed in this study was originally developed by Lian et al. (2005) and tailored specifically for college students. This instrument is utilized to evaluate the extent of academic burnout experienced by nursing students. It comprises 20 items distributed across three dimensions: behavioral misconduct, emotional exhaustion, and lack of personal accomplishment. It employs a five-point Likert scale, where scores range from “1” (completely disagree) to “5” (completely agree). The total score ranges from 20 to 100, with a score at least 60 indicating the presence of learning burnout; higher scores signify more severe burnout. Compared with other scales, the learning burnout scale used in this study, developed by Lian et al. (2005) based on a sample of Chinese college students, is more culturally and contextually appropriate for Chinese college students. In this study, the scale demonstrated excellent reliability with a Cronbach’s α coefficient of 0.970.

#### 2.2.5 Perceived Social Support Scale (PSSS)

Perceived Social Support Scale was adopted from the formulation by Zimet et al. (1991), translated by Jiang (2001), and revised by Yan (2006). This scale consists of 12 items, including three dimensions of friends’ support, family support, and other support. The scale uses a seven-point Likert scale scoring method, ranging from “1 to 7” to represent “extremely disagree” to “extremely agree.” The total score is the sum of all items and ranges between 12 and 84 higher total score indicates that an individual perceives more social support. Specifically, a total score of 61–84 indicates high social support, 37–60 indicates medium social support, and 12–36 indicates low social support. Compared with other scales, the PSSS measures the subjective perception of social support experienced by individuals, rather than objective support behaviors. Furthermore, its 12-item structure makes it concise and well-suited for use among college students. In this study, this scale has good reliability, with a Cronbach’s α coefficient of 0.962.

### 2.3 Statistical analysis

This study employed SPSS 22.0 for statistical analysis. A *P*-value less than 0.05 in both tails indicated statistical significance. Using *t*-test to explore the scores and significant differences of depression, self-efficacy, academic burnout, and social support among undergraduate students majoring in eldercare services with different demographic factors. Pearson correlation analysis was used to explore the correlations. Using PROCESS Version 4.0 of SPSS for mediation effect analysis, Model 4 for mediation analysis, and Model 8 for moderated mediation effect analysis. Tooking academic burnout as the dependent variable, depression as the independent variable, self-efficacy as the mediating variable, and social support as the moderating variable to include them in the model for analysis. A total of 5,000 samples were simulated using the bootstrap method. If the 95% bias-corrected confidence interval did not include 0, the results were significant. The test standard α was set at 0.05.

### 2.4 Ethical declaration

This research complies with ethical standards and has been approved by the Ethics Committee of Shenyang Medical College.

## 3 Results

### 3.1 Participants demographics

Among the 780 students, 150 were male (19.20%), and 630 were female. The ratio of male to female was quite different. There were 457 students under the age of 20 (58.60%), and 323 students aged 20 or above (41.40%). Six hundred and twelve students (78.50%) were from medical colleges, and 168 students (21.50%) were from comprehensive colleges. A total of 358 students (45.90%) were freshmen, and 422 students (54.10%) were sophomores or above. A total of 358 students (31.00%) were the only child in the family, and 538 students (53.80%) were not. A total of 444 students (56.90%) had fathers with educational attainment of junior high school level or below, and 336 students (43.10%) had fathers with educational attainment above the middle school level. A total of 471 students (60.40%) had mothers with educational attainment of junior high school level or below, and 309 students (39.60%) had mothers with educational attainment above the junior high school level. A total of 125 students (16.00%) received scholarships, and 655 students (84.00%) did not receive scholarships. A total of 381 students (48.80%) exercised for less than 30 min a day, and 399 students (51.20%) exercised for more than 30 min. A total of 713 students (91.40%) slept for less than 6 h, and 67 students (8.60%) slept for more than 6 h. Only 40 students (5.10%) smoked. 622 students (84.90%) were raised by both parents.

Independent samples t-tests were conducted to examine differences in depression, self-efficacy, learning burnout, and perceived social support across various demographic variables. First, the results indicated that male students, those aged ≥ 20, students from medical universities, senior students, non-children, individuals with less than 30 min of daily exercise, and smokers were more likely to experience higher levels of depression (*P* < 0.05). The Cohen’s d ranged from 0.491 to 0.500, which falls within the range of a medium effect according to conventional benchmarks. This suggests that the aforementioned demographic variables do exert some influence on depression, although the magnitude of this effect is moderate. Second, the analysis revealed that students aged < 20, only children, those whose parents had a high school education or higher, scholarship recipients, and those engaging in 30 min or more of daily exercise exhibited higher levels of self-efficacy (*P* < 0.05). The Cohen’s d ranged from 0.663 to 0.671, which falls within the range of a moderately large effect according to standard effect size conventions. This indicates that the aforementioned demographic variables have a notable influence on self-efficacy, with a relatively strong effect.

Third, the findings showed that male students, those aged 20 or older, students from medical colleges, non-only children, individuals whose parents had a high school education or lower, those not raised by their parents, non-scholarship recipients, individuals with less than 30 min of daily exercise, and smokers were more prone to learning burnout (*P* < 0.05). The Cohen’s d ranged from 0.582 to 0.590, which falls within the range of a moderately large effect according to standard effect size criteria. This indicates that the aforementioned demographic variables have a significant impact on learning burnout, with a strong effect. Fourth, the results indicated that female students, those under 20 years old, students from comprehensive colleges, freshmen, only children, individuals whose parents had a high school education or above, those raised by their parents, scholarship recipients, individuals with 30 min or more of daily exercise, and non-smokers reported higher levels of perceived social support (*P* < 0.05). The Cohen’s d ranged from 1.084 to 1.096, which falls within the range of a large effect size according to established effect size benchmarks. This indicates that the aforementioned demographic variables have a substantial impact on social support, with a strong effect. The detailed findings are presented in Tables 1, 2.

**TABLE 1:** Scores of depression, self-efficacy, academic burnout and social support.

Variables	*N* (%)	Depression	Self-efficacy	Academic burnout	Social support
		(M ± SD)	(M ± SD)	(M ± SD)	(M ± SD)
**Gender**					
Male	150 (19.20%)	17.72 ± 11.05**	27.40 ± 6.76	53.21 ± 12.50**	60.23 ± 14.52
Female	630 (80.80%)	15.13 ± 9.61	26.93 ± 6.70	50.33 ± 11.58	63.69 ± 12.77**
**Age**					
< 20	457 (58.60%)	14.30 ± 9.96	27.53 ± 7.02*	49.35 ± 12.18	64.84 ± 12.95**
≥ 20	323 (41.40%)	17.50 ± 9.64**	26.30 ± 6.19	53.05 ± 10.92**	60.45 ± 13.11
**School**					
Medical universities	612 (78.50%)	16.41 ± 9.94**	26.84 ± 6.50	51.71 ± 11.48**	62.36 ± 13.26
Comprehensive universities	168 (21.50%)	12.76 ± 9.47	27.70 ± 7.41	47.88 ± 12.50	65.42 ± 12.67**
**Grade**					
Freshman	358 (45.90%)	14.70 ± 9.63	26.62 ± 6.98	50.31 ± 11.90	64.12 ± 12.82*
Sophomore	422 (54.10%)	16.42 ± 10.15*	27.36 ± 6.46	51.36 ± 11.72	62.09 ± 13.43
**Only child**					
Yes	242 (31.00%)	13.83 ± 9.87	28.06 ± 7.02**	49.04 ± 13.16	65.47 ± 13.34**
No	538 (69.00%)	16.43 ± 9.88**	26.56 ± 6.52	51.71 ± 11.06**	61.92 ± 12.98
**Parents’ educational level**					
Below high school	388 (49.74%)	17.14 ± 9.84**	25.94 ± 6.55	52.90 ± 11.09**	60.78 ± 13.05
High school or above	392 (50.26%)	14.09 ± 9.79	28.10 ± 6.70**	48.85 ± 12.16	65.21 ± 12.91**
**Parenting style**					
Parent	622 (84.90%)	15.40 ± 9.96	27.08 ± 6.75	50.46 ± 12.00	63.61 ± 13.13**
Others	118 (15.10%)	16.92 ± 9.79	26.72 ± 6.48	53.25 ± 10.38*	59.70 ± 13.04
**Scholarship**					
Yes	125 (16.00%)	14.85 ± 9.73	28.48 ± 6.20**	48.51 ± 11.51	65.66 ± 11.95*
No	655 (84.00%0	15.78 ± 9.99	26.75 ± 6.77	51.33 ± 11.82*	62.52 ± 13.36
**Exercise duration**					
< 30 min/day	381 (48.80%)	16.99 ± 10.03**	26.45 ± 6.50	52.82 ± 11.08**	61.66 ± 13.13
≥ 30 min/day	399 (51.20%)	14.33 ± 9.70	27.57 ± 6.87*	49.03 ± 12.19	64.32 ± 13.11**
**Sleep duration**					
< 6 h/day	713 (91.40%)	15.79 ± 9.88	26.88 ± 6.69	51.00 ± 11.74	62.73 ± 12.90
≥ 6 h/day	67 (8.60%)	13.85 ± 10.50	28.55 ± 6.81	49.61 ± 12.50	66.18 ± 15.64
**Smoking**					
Yes	40 (5.10%)	21.33 ± 12.40**	27.30 ± 7.08	57.00 ± 12.06**	55.05 ± 17.55
No	740 (94.90%)	15.32 ± 9.71	27.01 ± 6.69	50.55 ± 11.71	63.45 ± 12.78**

*P < 0.05. **P < 0.01.

**TABLE 2:** Cohen’s d of depression, self-efficacy, academic burnout and social support under different demographic statistics.

Variables	Depression	Self-efficacy	Academic burnout	Social support
Gender	0.495	0.671	0.588	1.094
Age	0.491	0.669	0.584	1.085
School	0.492	0.670	0.585	1.094
Grade	0.500	0.670	0.590	1.096
Only child	0.494	0.668	0.587	1.091
Parents’ educational level	0.492	0.663	0.582	1.084
Parenting style	0.497	0.671	0.589	1.093
Scholarship	0.497	0.668	0.588	1.095
Exercise duration	0.493	0.669	0.583	1.094
Sleep duration	0.497	0.670	0.590	1.096
Smoking	0.493	0.671	0.586	1.089

### 3.2 Correlation between academic burnout, self-efficacy, depression and social support

Pearson correlation analysis was conducted on depression, self-efficacy, academic burnout, and social support. The results showed that depression was significantly negatively correlated with self-efficacy (*r* = −0.385, *P* < 0.01), depression was significantly positively correlated with academic burnout (*r* = 0.646, *P* < 0.01), and self-efficacy was significantly negatively correlated with academic burnout (*r* = −0.486, *P* < 0.01). The details are presented in Table 3.

**TABLE 3:** Correlation analysis among depression, self-efficacy, academic burnout and social support.

Variables	①	②	③	④	⑤	⑥	⑦	⑧	⑨	⑩	⑪	⑫	⑬	⑭
**Depression**	1													
Depressed affect	0.945**	1												
Somatic and retarded activity	0.924**	0.873**	1											
Positive affect	0.638**	0.417**	0.400**	1										
Interpersonal	0.821**	0.806**	0.775**	0.372**	1									
**Self-efficacy**	−0.385**	−0.301**	−0.282**	−0.471**	−0.221**	1								
**Academic burnout**	0.646**	0.584**	0.580**	0.488**	0.489**	−0.486**	1							
Behavioral misconduct	0.569**	0.516**	0.505**	0.437**	0.419**	−0.485**	0.904**	1						
Emotional exhaustion	0.575**	0.533**	0.534**	0.387**	0.450**	−0.339**	0.932**	0.721**	1					
Lack of personal accomplishment	0.558**	0.457**	0.457**	0.564**	0.401**	−0.606**	0.679**	0.620**	0.465**	1				
**Social support**	−0.563**	−0.453**	−0.484**	−0.549**	−0.419**	0.446**	−0.473**	−0.455**	−0.335**	−0.612**	1			
Family support	−0.534**	−0.434**	−0.460**	−0.514**	−0.392**	0.403**	−0.446**	−0.420**	−0.323**	−0.571**	0.941**	1		
Friend support	−0.523**	−0.411**	−0.457**	−0.516**	−0.398**	0.383**	−0.407**	−0.384**	−0.289**	−0.541**	0.892**	0.810**	1	
Other support	−0.518**	−0.418**	−0.442**	−0.509**	−0.387**	0.447**	−0.454**	−0.449**	−0.314**	−0.587**	0.946**	0.805**	0.774**	1

**P < 0.01. ① depression; ② depressed affect; ③ somatic and retarded activity; ④ positive affect; ⑤ interpersonal; ⑥ self-efficacy; ⑦ academic burnout; ⑧ behavioral misconduct; ⑨ emotional exhaustion; ⑩ lack of personal accomplishment; ⑪ social support; ⑫ family support; ⑬ friend support; ⑭ other support.

### 3.3 The mediating effect and regression analysis of self-efficacy

**FIGURE 2:**
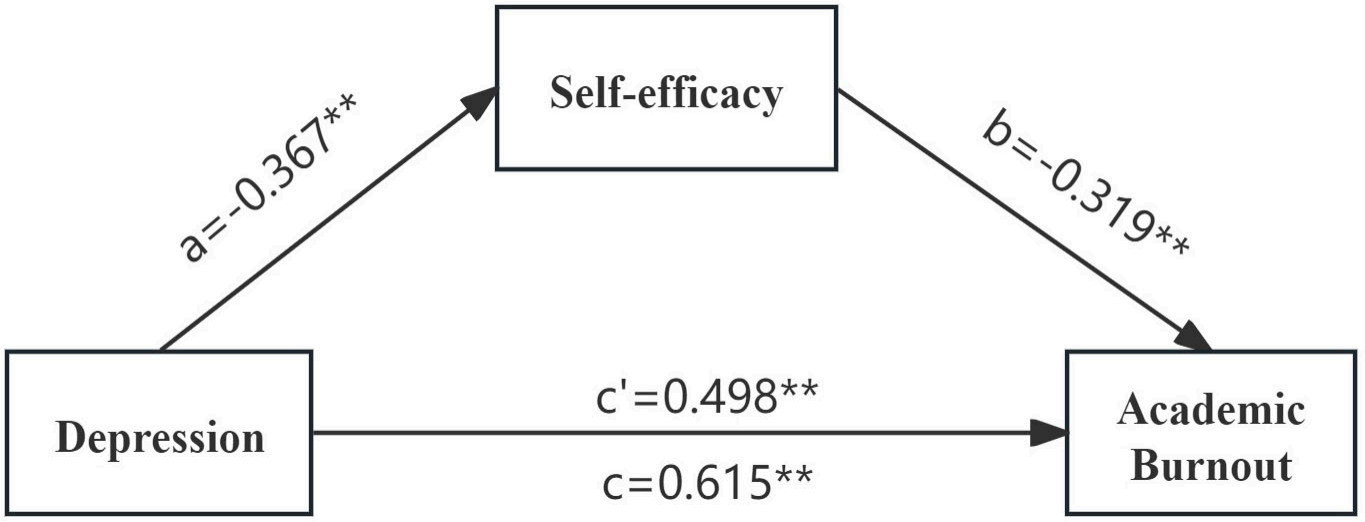
Moderated mediating effect model diagram. **P < 0.01.

The model 4 in the process macro compiled by Hayes was used to test the mediating effect of self-efficacy between depression and academic burnout. The results are shown in Tables 4, 5. The negative effect of daily exercise time on students’ academic burnout was significant (β = −0.075, *P* < 0.05). The positive effect of depression on the academic burnout of undergraduate students majoring in eldercare services was significant (β = 0.615, *P* < 0.001). After adding the mediating variable of self-efficacy, the positive predictive effect of depression on academic burnout remained significant (β = 0.498, *P* < 0.01), and depression showed a significant negative effect on self-efficacy (β = −0.367, *P* < 0.01), while self-efficacy also showed a significant negative effect on academic burnout (β = −0.319, *P* < 0.01). The research results showed that the total effect value was 0.731, the direct effect value was 0.592, and the mediating effect value of self-efficacy between depression and academic burnout was 0.139. The direct effect accounted for 80.98% of the total effect, and the mediating effect accounted for 19.02% of the total effect, with a 95% confidence interval of 0.100–0.183, not including zero, indicating that self-efficacy can directly and indirectly predict academic burnout (Figure 2).

**TABLE 4:** The mediating effect of self-efficacy between depression and academic burnout.

Predictors	Model 1			Model 2			Model 3		
	Academic burnout			Self-efficacy			Academic burnout		
	*B*	t	95% CI	*B*	t	95% CI	*B*	t	95% CI
Gender	−0.022	−0.771	(−0.117, 0.051)	−0.066	−1.876	(−0.229, 0.005)	−0.043	−1.635	(−0.142, 0.013)
Age	0.040	1.414	(−0.018, 0.113)	−0.032	−0.922	(−0.135, 0.049)	0.029	1.142	(−0.025, 0.096)
School	−0.032	−1.142	(−0.124, 0.033)	−0.003	−0.100	(−0.115, 0.104)	−0.033	−1.284	(−0.119, 0.025)
Only child	0.004	0.139	(−0.067, 0.077)	−0.011	−0.310	(−0.117, 0.085)	0.001	0.019	(−0.066, 0.067)
Parents’ educational level	−0.059	−2.019*	(−0.137, −0.002)	0.091	2.530*	(0.027, 0.216)	−0.030	−1.109	(−0.098, 0.027)
Scholarship	0.065	2.372*	(0.018, 0.190)	−0.081	−2.410*	(−0.268, −0.027)	0.039	1.543	(−0.017,0.142)
Exercise duration	−0.068	−2.435	(−0.145, −0.016)	0.010	0.284	(−0.077, 0.103)	−0.065	−2.525*	(−0.136, −0.017)
Smoking	−0.038	−1.337	(−0.250, 0.048)	−0.034	−0.965	(−0.311, 0.106)	−0.049	−1.864	(−0.267, 0.007)
Parenting	0.041	1.529	(−0.019, 0.155)	0.002	0.072	(−0.117, 0.126)	0.042	1.693	(−0.011, 0.149)
Depression	0.615	21.824**	(0.665, 0.796)	−0.367	−10.567**	(−0.587, −0.403)	0.498	17.946**	(0.527, 0.656)
Self-efficacy	–	–	–	–	–	–	−0.319	−11.850**	(−0.327, −0.234)
*R* ^2^	0.452**			0.168**			0.536**		
*F*	63.327			15.549			80.771		

*P < 0.05. **P < 0.01.

**TABLE 5:** Analysis of total effect, direct effect, and mediating effect.

Effect type	B	Boot SE	Boot LLCI	Boot ULCI	Proportion of effects
Total effect	0.731	0.034	0.665	0.796	–
Direct effect	0.592	0.033	0.527	0.656	80.98%
Mediation effect of self-efficacy	0.139	0.021	0.100	0.183	19.02%

### 3.4 The moderating effect of social support

**FIGURE 3:**
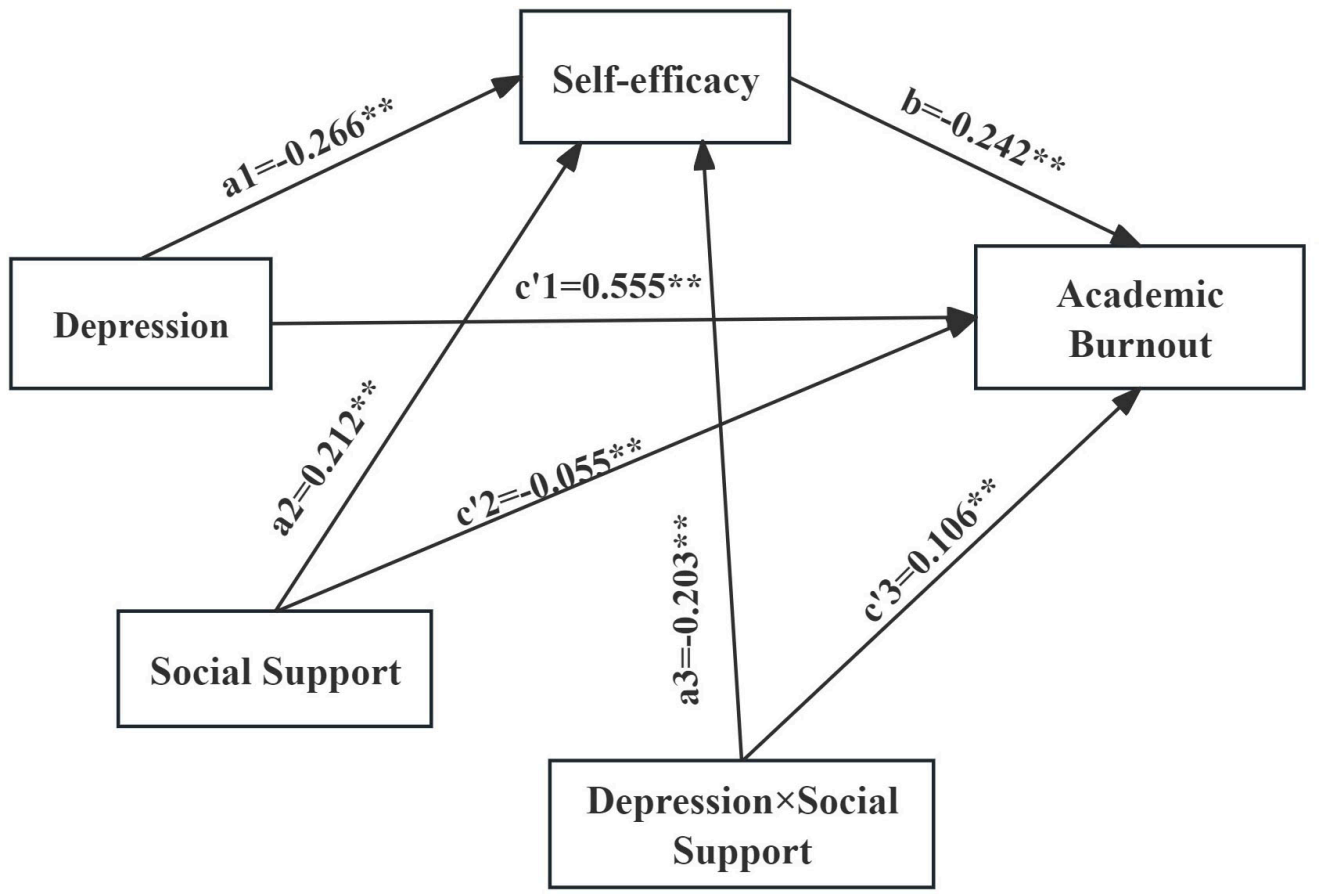
Moderated mediating effect model diagram. **P < 0.01.

**FIGURE 4:**
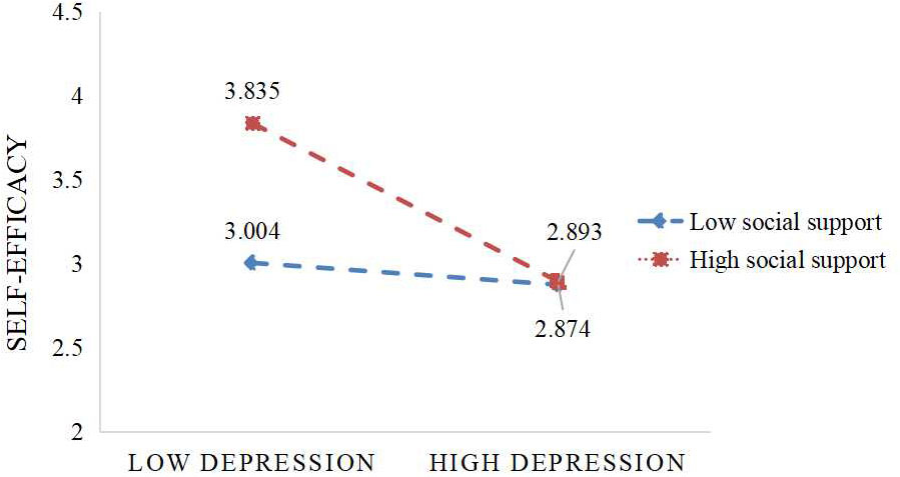
The moderating role of social support in the relationship between depression and self-efficacy.

**FIGURE 5:**
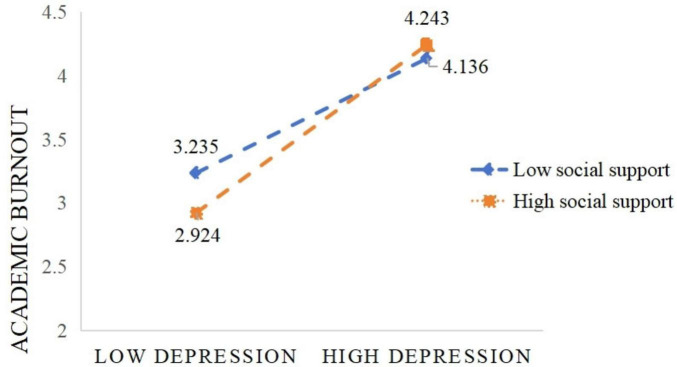
The moderating role of social support in the relationship between depression and academic burnout.

The regulatory effect of social support was examined using Model 8 from the “process” macro developed by Hayes. The results are presented in Table 6 and Figure 3. After including social support in the model, the interaction term between depression and social support showed a significant negative predictive effect on self-efficacy (β = −0.203, *P* < 0.01), and a significant positive effect on academic burnout (β = 0.106, *P* < 0.01). This indicates that social support can play a regulatory role in the prediction of self-efficacy by depression, and also in the prediction of academic burnout by depression. Through simple slope analysis, the regulatory effect of social support between depression and self-efficacy is shown in Figure 4. At high levels of social support, as the depression level increases, the self-efficacy level shows a downward trend (*B_*simple*_* = −0.491, *P* < 0.01). The regulatory effect of social support between depression and academic burnout is shown in Figure 5. At both high and low levels of social support, as the depression level increases, the academic burnout level shows an upward trend (*B_*simple*_* = 0.670, *P* < 0.01; *B*_*simple*_ = 0.440, *P* < 0.001). Compared to the low group, the positive effect of the high group is stronger. With the increase in the level of social support, the effect of depression on self-efficacy shows a gradually increasing trend, and the mediating effect of self-efficacy between depression and academic burnout also shows an upward trend, as shown in Table 7.

**TABLE 6:** Test of moderating effect of social support.

Predictors	Model 1			Model 2		
	Self-efficacy			Academic burnout		
	*B*	t	95% CI	*B*	t	95% CI
Gender	−0.107	−1.909	(−0.218, 0.003)	−0.066	−1.700	(−0.143, 0.01)
Age	−0.005	−0.108	(−0.091, 0.082)	0.026	0.838	(−0.034, 0.085)
School	−0.021	−0.396	(−0.124, 0.082)	−0.041	−1.116	(−0.112, 0.031)
Only child	0.008	0.171	(−0.087, 0.103)	−0.007	−0.198	(−0.072, 0.059)
Parents’ educational level	0.085	1.887	(−0.004, 0.174)	−0.029	−0.925	(−0.091, 0.033)
Scholarship	−0.107	−1.85	(−0.220, 0.007)	0.058	1.439	(−0.021, 0.136)
Exercise duration	0.009	0.213	(−0.076, 0.094)	−0.077	−2.592*	(−0.136, −0.019)
Smoking	−0.136	−1.358	(−0.333, 0.061)	−0.127	−1.839	(−0.263, 0.009)
Parenting	0.058	0.988	(−0.057, 0.172)	0.053	1.306	(−0.027, 0.132)
Depression	−0.266	−5.189**	(−0.367, −0.166)	0.555	15.388**	(0.484, 0.626)
Social support	0.212	9.086**	(0.166, 0.258)	−0.055	−3.234**	(−0.088, −0.022)
Self-efficacy	–	–	–	−0.242	−9.691**	(−0.291, −0.193)
Depression × social support	−0.203	−5.439**	(−0.276, −0.129)	0.106	4.031**	(0.054, 0.157)
*R* ^2^	0.269**			0.550**		
*F*	23.519			72.118		

*P < 0.05, **P < 0.01.

**TABLE 7:** Moderated mediating effect.

Moderated mediation analysis	Indicators	Effect	Boot SE	Boot LLCI	Boot ULCI
Moderated mediating effect	eff1 (M−1SD)	0.011	0.021	−0.030	0.056
	eff2 (M)	0.065	0.017	0.035	0.103
	eff2 (M + 1SD)	0.119	0.023	0.080	0.170
The contrast of moderated mediating effects	eff2−eff1	0.054	0.014	0.029	0.085
	eff3−eff1	0.108	0.028	0.058	0.169
	eff3−eff2	0.054	0.014	0.029	0.085

## 4 Discussion

### 4.1 Detection rate of academic burnout among undergraduate students majoring in eldercare services

Currently, the elderly care industry is predominantly staffed by female workers, which aligns with the observation that the proportion of female participants in this study was significantly higher than that of male participants. The findings of this study are therefore representative and applicable to the broader elderly care industry. The study revealed that the detection rate of academic burnout among undergraduate students majoring in elderly services was 26.92%, which is lower than the 49% reported by Thun-Hohenstein et al. (2021) and the 44% academic burnout rate observed among medical students globally (Frajerman et al., 2019). This may be related to differences in survey schools and survey scales. Compared with Vasconcelos’s 20.0% detection rate of academic burnout among Brazilian nursing students and Kong’s 23.0% research results, this difference can be attributed to the larger sample size used in this study, which reduced sampling errors, improved the representativeness and accuracy of the research results, and thus more comprehensively revealed the detection rate of academic burnout among undergraduate students majoring in eldercare services (Kong et al., 2023; Vasconcelos et al., 2020).

### 4.2 Depression has a negative impact on academic burnout

Among the undergraduate students majoring in eldercare services in this study, depression, self-efficacy, academic burnout, and social support were significantly correlated pairwise. Depression was significantly negatively correlated with self-efficacy and social support, meaning that as depression levels increased, students’ self-efficacy significantly decreased. Depression was significantly positively correlated with academic burnout, meaning that as depression levels increased, academic burnout also increased, which is consistent with the results of related studies, and the hypothesis one was established (Liu et al., 2024; Meier and Kim, 2022). Academic burnout is a long-term, accumulated psychological state, manifested as emotional exhaustion, decreased academic efficacy, and negative attitudes toward learning. These characteristics are very similar to the core symptoms of depression. Students majoring in eldercare services frequently encounter aging, diseases, and end-of-life care scenarios regularly, which leads to the loss of interest in studies and life, and subsequently, the emergence of depression. In this situation, students’ learning efficiency may decrease, their learning state may become negative, and they may develop academic burnout. Therefore, it is crucial for students majoring in eldercare services to maintain a positive mindset while enhancing self-efficacy and increasing social support, which can effectively reduce academic burnout.

### 4.3 The mediating role of self-efficacy between depression and academic burnout

The mediation effect test shows that self-efficacy plays a partial mediating role between depression and academic burnout, indicating that depression not only directly predicts learning burnout but also can influence academic burnout through self-efficacy. This is consistent with the previous research results, and research hypothesis two is confirmed (Zhu et al., 2023). The theory of self-efficacy holds that self-efficacy is a protective factor against stress, which will affect an individual’s attitude and behavior in specific situations, thereby influencing their effort and perseverance. Depressive emotions may lead students to have a negative evaluation of academic goals; this cognitive bias further reduces their effort level, forming a vicious cycle of “low efficacy-high burnout.” Undergraduate students with depressive emotions have lower learning adaptability and are more likely to experience setbacks, despair, and hopelessness (Zhang et al., 2022), which may cause a decrease in self-efficacy, leading students to believe that they cannot complete learning tasks. When students believe that they cannot effectively utilize their energy, they will have negative expectations for good academic performance. Therefore, students should actively adopt effective strategies to enhance self-efficacy, thereby maintaining a good mindset, having high-level learning motivation, seeking social support, participating in sports activities, learning relaxation techniques, etc., to overcome negative emotions, and thereby reducing the negative impact of depressive emotions on academic burnout.

### 4.4 The moderating effect of social support on depression, academic burnout, and self-efficacy

The adjusted mediating effect test shows that the product term of depression and social support can significantly predict self-efficacy. This indicates that social support can effectively regulate the impact of depression on self-efficacy, thus confirming hypothesis three. Specifically, when students receive a high level of social support, the negative impact of depression on self-efficacy will be reduced; the product term of depression and social support can also significantly predict academic burnout, indicating that social support can also effectively regulate the impact of depression on academic burnout, thus confirming hypothesis four. When students have a lower level of social support, the impact of depression on academic burnout will increase. That is to say, when undergraduate students majoring in eldercare services are in a state of depression, it is appropriate to increase the intensity of social support to reduce the adverse effects of depression on academic burnout, which is consistent with the existing research results (Cheng et al., 2020; Meier and Kim, 2022). This is also consistent with the buffering hypothesis, which holds that social support provides an increase in coping ability to alleviate the negative effects of stress (Yuan et al., 2025). Social support, as an important psychological resource, reduces the negative impact of academic burnout on depression through providing emotional comfort, information support, and practical assistance. Due to the long-term impact of depression, students with a high degree of academic burnout may exhibit avoidance behavior in learning, show a cold attitude toward things, reduce social interaction, and thus have limited access to actual support (Kim et al., 2018). With the decrease in social support and the increase in isolation, students’ participation in academic and daily activities may decline, thereby forming a cycle of negative cognition and behavior reinforcement. Therefore, to alleviate the impact of depression on the academic burnout of undergraduate students majoring in eldercare services, in the process of mental health intervention, attention should be paid to improving their social support level, especially from family, friends, and school.

## 5 Conclusion

This study constructed a moderated mediating model to explore the influencing factors and mechanism of academic burnout among undergraduate students majoring in eldercare services. The results showed that depression of undergraduate students majoring in eldercare services could directly affect academic burnout. Self-efficacy played a partial mediating role in the relationship between academic burnout and depression, and social support played a moderating role between depression and self-efficacy as well as between depression and academic burnout. Therefore, improving the social support level of undergraduate students majoring in eldercare services can help alleviate their academic burnout levels and promote their psychological health development.

## 6 Limitations

First, this study employed a cross-sectional design, which limits the ability to establish causal relationships between depression, self-efficacy, social support, and academic burnout. Second, the sample size was relatively small, with data collected from only 957 students, which may have affected the representativeness of the study population. Third, the study did not account for other potential factors associated with academic burnout, such as academic pressure, career identity, and psychological resilience.

Future research will consider longitudinal designs to explore potential causal relationships, expand the sample size, and incorporate additional relevant variables. Such improvements would contribute to a more comprehensive understanding of how psychological and social factors influence academic burnout among students.

## Data Availability

The raw data supporting the conclusions of this article will be made available by the authors, without undue reservation.
