# Correction: The relationship between depression and academic burnout among undergraduate students majoring in eldercare services: a moderated mediation model

**DOI:** 10.3389/fpsyg.2025.1741272

**Published:** 2025-11-19

**Authors:** Yingjie Lin, Siyu Pan, Jianyao He, Qi Cheng, Hongmin Wei, Li Zhao

**Affiliations:** 1 School of Geriatrics and Elderly Care Industry, Shenyang Medical College, Shenyang, Liaoning, China; 2 Division of Academic Affairs, Kunming Medical University, Kunming, Yunnan, China; 3 School of Health Industry, Anshan Normal University, Anshan, Liaoning, China; 4 Department of Nursing, Beijing Health Vocational College, Beijing, China

**Keywords:** depression, self-efficacy, academic burnout, social support, moderated mediation

Affiliation 1 School of Geriatrics and Elderly Care Industry, Shenyang Medical College, Shenyang, Liaoning, China was inadvertently omitted for author Li Zhao. This affiliation has now been added for author Li Zhao.

The original version of this article has been updated.

